# Detection of a Potential New *Bartonella* Species “Candidatus *Bartonella rondoniensis*” in Human Biting Kissing Bugs (Reduviidae; Triatominae)

**DOI:** 10.1371/journal.pntd.0005297

**Published:** 2017-01-17

**Authors:** Maureen Laroche, Jean-Michel Berenger, Oleg Mediannikov, Didier Raoult, Philippe Parola

**Affiliations:** URMITE, Aix Marseille Université, UM63, CNRS 7278, IRD 198, INSERM 1095, IHU—Méditerranée Infection, 19–21 Boulevard Jean Moulin, Marseille; Universidad de Buenos Aires, ARGENTINA

## Abstract

**Background:**

Among the Reduviidae family, triatomines are giant blood-sucking bugs. They are well known in Central and South America where they transmit *Trypanosoma cruzi* to mammals, including humans, through their feces. This parasitic protozoan is the causative agent of Chagas disease, a major public health issue in endemic areas. Because of the medical and economic impact of Chagas disease, the presence of other arthropod-borne pathogens in triatomines was rarely investigated.

**Methodology/Principal findings:**

In this study, seven triatomines species involved in the transmission of *T*. *cruzi* were molecularly screened for the presence of known pathogens generally associated with arthropods, such as *Rickettsia*, *Bartonella*, *Anaplasmataceae*, *Borrelia* species and *Coxiella burnetii*. Of all included triatomine species, only *Eratyrus mucronatus* specimens tested positive for *Bartonella* species for 56% of tested samples. A new genotype of *Bartonella* spp. was detected in 13/23 *Eratyrus mucronatus* specimens, an important vector of *T*. *cruzi* to humans. This bacterium was further characterized by sequencing fragments of the *ftsZ*, *gltA* and *rpoB* genes. Depending on the targeted gene, this agent shares 84% to 91% of identity with *B*. *bacilliformis*, the agent of Carrion’s disease, a deadly sandfly-borne infectious disease endemic in South America. It is also closely related to animal pathogens such as *B*. *bovis and B*. *chomelii*.

**Conclusions:**

As *E*. *mucronatus* is an invasive species that occasionally feeds on humans, the presence of potentially pathogenic *Bartonella*-infected bugs could present another risk for human health, along with the *T*. *cruzi* issue.

## Author Summary

Triatomines are hematophagous insects including vectors of *T*. *cruzi*, the agent of Chagas disease, a huge public health issue, especially in South America. Whether these arthropods carry other pathogenic microorganisms is currently unknown. We investigated the presence of different arthropod-borne pathogens, including *Bartonella* spp., by quantitative PCR. *Bartonella* species were identified using *ftsZ*, *gltA* and *rpoB* gene sequencing and a new genotype of *Bartonella* spp. was detected in *Eratyrus mucronatus* specimens, an important vector of *T*. *cruzi* to humans. This agent is closely related to several human and animal pathogens. Depending on the gene fragment used, this agent shares 84% to 91% of identity with *B*. *bacilliformis*, the agent of the deadly Carrion’s disease. The possibility of transmission of potentially pathogenic bacteria could be an additional threat to human health since *E*. *mucronatus* bugs are more and more anthropophilic.

## Introduction

Triatomine bugs (order Hemiptera, family Reduviidae, subfamily Triatominae) are blood-sucking arthropods (“kissing bugs”), most of which can feed both on animals and humans. All stages from first instar to male and female adults are strictly hematophagous and responsible for a relatively large blood intake due to their large size. They are mainly sylvatic and feed on small wild mammals but can also feed on birds and bats [1]. Triatomines occupy diverse natural ecotopes, such as mammal and bird nests, hollow trees, caves and rock fissures [2], but also rural environments, as they can prosper in crevices in houses [1]. These arthropods are distributed world-wide but the vast majority of the 140 recognized species is found in the Americas [3]. They are particularly well studied in South America, where they transmit an endemic flagellate pathogen, *T*. *cruzi*, the etiological agent of Chagas disease [1]. Also known as the American trypanosomiasis, Chagas disease is a neglected tropical disease, the first human parasitic disease in the endemic areas. *T*. *cruzi* is transmitted through the feces of infected kissing bugs, causing heart failure 10 to 30 years post-infection for almost 30% of individuals [4].

Because of the public health impact of Chagas disease, studies on kissing bugs are mainly focused on this theme. As a matter of fact, the presence of other human pathogens was never described in the hundred years that it has been known that kissing bugs could transmit pathogens. Only the presence of *Wolbachia* and *Arsenophonus* species was investigated based on the fact that these obligate intracellular bacteria are known to be endosymbionts of many arthropods [5,6]. The presence of zoopathogenic arthropod-borne viruses was also investigated. To our knowledge, there is no report of pathogen detection in dejections or in triatomines themselves, except for *T*. *cruzi*, although *Arsenophonus nasoniae* was once reported to be detected in an eschar of a human [7]. Regarding viruses, two have been described in these bugs. *Triatoma virus* is reported as strictly entomopathogenic, particularly for its principal host, *Triatoma infestans* [8], while *African swine fever virus* was detected in *Triatoma gerstaeckeri* but not transmitted to pigs [9].

French Guiana is an 84,000 km^2^ overseas department and region of France bordered by Brazil and Suriname. Because of its many different ecosystems, particularly a dense rainforest, French Guiana is a biodiversity hotspot and one of the 21 areas where Chagas disease is endemic [10]. Among the 27 described species of triatomines in this area, many are invasive species. That is to say that many of them temporarily leave their sylvatic or peridomestic dwellings in order to invade houses. The main vector community of French Guiana comprises highly anthropophilic bugs belonging to the *Panstrongylus*, *Rhodnius* and *Eratyrus* genera [10]. They accidentally feed on humans [11] and also on potentially infected animals since they easily feed on domestic animals or wild mammals.

Aiming to add to knowledge regarding bacteria and triatomine association, we screened seven species of triatomines bugs from French Guiana by molecular biology for the presence of arthropod-borne bacteria such as *Rickettsia*, *Bartonella*, *Borrelia*, *Anaplasma*, *Wolbachia*, *Ehrlichia* species and *Coxiella burnetii*.

## Methods

### Triatomine collection, identification and selection

**Fig 1:**
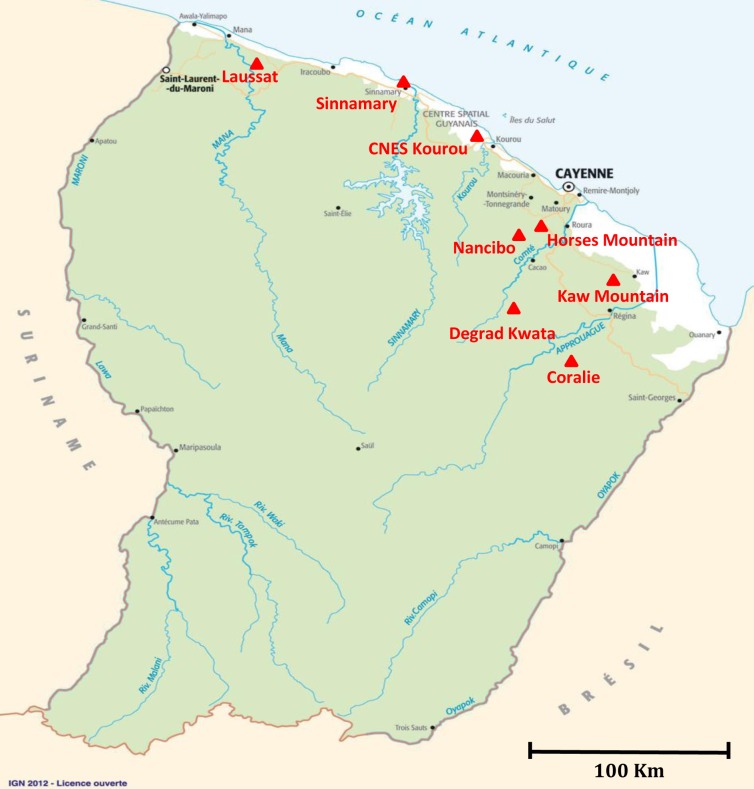
Distribution of sampling areas in French Guiana. Exact sampling sites are indicated by a triangle.

Triatomine specimens were collected in French Guiana from 1991 to 2013 using light traps or interception traps by one of the authors (JMB) and by the Société Entomologique Antilles-Guyane (SEAG) as part of an inventory of French Guiana’s insects. Triatomines were caught in forests (Horses Mountains, Kaw Mountains), peridomiciliary areas (Degrad Kwata, Kaw Mountains, Nancibo) or urban areas (Sinnamary, Kourou savannah) as displayed in Fig 1.

All triatomine specimens were morphological identified with the Bérenger et al. morphological key [12] and kept dried as insect collections. Seven *T*.*cruzi* vectors were included in this study: *Rhodnius prolixus* (n = 10), *Rh*. *pictipes* (n = 10), *Rh*. *robustus* (n = 10), *Triatoma infestans* (n = 10), *Panstrongylus geniculatus* (n = 10), *P*. *rufotuberculatus (n = 4)*, *Eratyrus mucronatus (n = 23)*.

### DNA extraction

Dried triatomines were rinsed in sterile water and air-dried on filter paper before cutting lengthwise in two equal halves, using a sterile surgical blade for each specimen. One half and the legs were stored at -20°C as a backup sample and the other legless half was selected for molecular analyses. Each half triatomine was crushed with a sterile pestle in 400 μL of a G2 buffer solution containing 40 μM of proteinase K (Qiagen) and incubated at 56°C overnight. After 1 minute of centrifugation at 7000 x g, 200 μL of the supernatant was then collected prior to DNA extraction. *Triatominae* genomic DNA was individually extracted using the EZ1 DNA tissue extraction kit (Qiagen, Hilden, Germany) according to the manufacturer’s instructions. *Triatominae* DNAs were then eluted in 100 μL of Tris EDTA (TE) buffer using the DNA extracting EZ1 Advanced XL Robot (Qiagen) as previously described [13]. DNAs were either immediately used or stored at -20°C until used for molecular analysis. The DNA extracting EZI Advanced XL Robot was disinfected after each batch of extraction as per the manufacturer recommendations to avoid cross-contamination.

### Molecular analysis

DNA samples were individually tested by genus-specific PCR using primers and probes targeting specific sequences of *Bartonella* spp., but also *Rickettsia* spp., *Coxiella burnetii*, *Borrelia* spp., and all Anaplasmataceae species [14] as previously described [15] (Table 1). Real-time quantitative PCR (qPCR) was carried out according to the manufacturer’s protocol using a CFX Connect Real-Time PCR Detection System (Bio-rad, Hercules, CA, USA) with the Eurogentec Takyon qPCR kit (Eurogentec, Seraing, Belgium).

**Table 1:** Sequences of qPCR primers used to investigate the presence of pathogens’ DNA in the *E*. *mucronatus* samples. F: forward primer, R: reverse primer, P: qPCR probe.

Target organism	Target gene	Primer’s name	Sequence (5’-3’)
***Bartonella* spp.**	Intergenic spacer	IT2_F	GGGGCCGTAGCTCAGCTG
		ITS2_R	TGAATATATCTTCTCTTCACAATTTC
		ITS2_P	6FAM-CGATCCCGTCCGGCTCCACCA
***Rickettsia* spp.**	*gltA*	gltA_F	GTGAATGAAAGATTACACTATTTAT
		gltA_R	GTATCTTAGCAATCATTCTAATAGC
		gltA_P	6FAM-CTATTATGCTTGCGGCTGTCGGTTC
***Coxiella burnetii***	*IS30A*	ITS30A_F	CGCTGACCTACAGAAATATGTCC
		ITS30A_R	GGGGTAAGTAAATAATACCTTCTGG
		ITS30A_P	6FAM-CATGAAGCGATTTATCAATACGTGTATGC
***Borrelia* spp.**	*16S*	16S_F	AGCCTTTAAAGCTTCGCTTGTAG
		16S_R	GCCTCCCGTAGGAGTCTGG
		16S_P	6FAM- CCGGCCTGAGAGGGTGAACGG
***Anaplasmataceae***	23S	23S_F	TGACAGCGTACCTTTTGCAT
		23S_R	GTAACAGGTTCGGTCCTCCA
		23S_P	6FAM- GGATTAGACCCGAAACCAAG

*Bartonella elizabethae*, *Rickettsia montanensis*, *Coxiella burnetii*, *Anaplasma phagocytophilum* and *Borrelia crocidurae* DNAs were used as positive qPCR controls for the primers and probe targeting respectively all *Bartonella*, *Rickettsia*, *Coxiella burnetii* and *Borrelia* species.

DNAs were tested at different concentrations to avoid PCR inhibition. For each run, a PCR mix without DNA was used as negative control. Standard PCR targeting a 710 bp region of the invertebrate *cytochrome oxidase I* (*COI*) gene was performed on PCR negative triatomines to control DNA extraction.

### Sequencing and GenBank accession numbers

DNA samples that were positive with *Bartonella*-qPCR were submitted to conventional PCR amplification using a Bio-Rad Thermocycler (Bio-Rad Laboratories, Hercules, CA) prior to sequencing. For *Bartonella* species identification, primers targeting *Bartonella rpoB*, *gltA* and *ftsZ* genes fragments were used as previously described [16]. DNA from *Bartonella elizabethae* served as PCR positive control and mixture without DNA as negative control. The cycling protocol consisted of 15 min at 95°C followed by 35 cycles of denaturing at 95°C for 30 s, annealing at 50°C for 30 s (58°C for *rpoB* gene), extension 1 min at 72°C, followed by a final cycle of 1 min at 72°C and sampling held at 4°C. Amplification products were separated by electrophoresis through a 1.5% agarose-tris-borate-EDTA gel containing ethidium bromide. PCR products were sequenced using a Big Dye Terminator kit and an ABI PRISM 3130 Genetic Analyser (Applied BioSystems, Courtabeauf, France). The sequences were analyzed using the ABI PRISM DNA Sequencing Analysis software version 3.0 (Applied BioSystems) and compared to sequences available in the GenBank database using the BLAST algorithm (http://blast.ncbi.nlm.nih.gov/Blast.cgi). The partial sequences of *ftsZ* and *rpoB* genes of *Bartonella* amplified from the sample *EmG01* are available in GenBank at #KX377404 and #KX377405.

### Phylogenic analysis

Phylogeny of the detected *Bartonella* with other members of the *Bartonella* genus was established with TOPALi 2.5 software (Biomathematics and Statistics Scotland, Edinburgh, UK). Available sequences of *ftsZ*, *gltA* and *rpoB* genes of validated *Bartonella* species were retrieved from the National Center for Biotechnology Information (NCBI) based on the results of the BLAST program. Multiple sequence alignment was performed with the ClustalW multiple sequence alignment program, which is included in the BioEdit software.

## Results

### Triatominae collection

**Fig 2:**
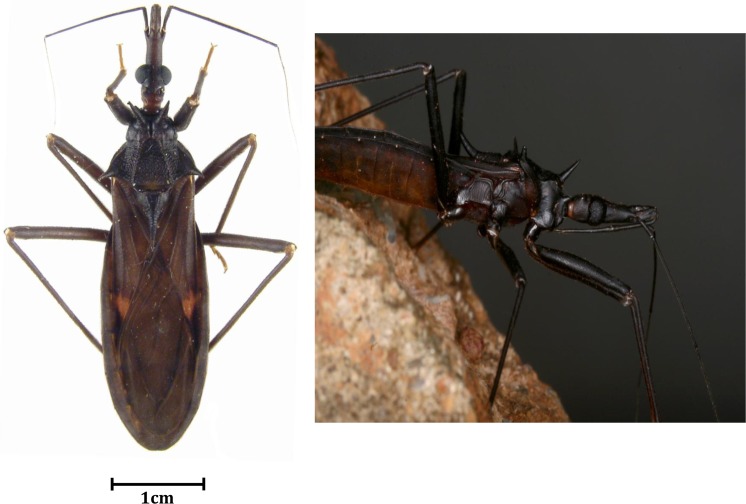
Pictures of alive and dead *Eratyrus mucronatus* in its environment and dried on paper.

Triatomines were collected in eight different localities in French Guiana with no selection regarding species and sex (convenient sampling). Among the triatomines collected, *Eratyrus mucronatus* (Fig 2) accounted for 20% of catches and 29.8% of the specimens analyzed. Details related to collection, such as sampling area and triatomines’ sex, are indicated in Table 2. Of 23 *E*. *mucronatus* samples, 22 (95.6%) were male. Further details regarding other collected species have been listed elsewhere [12].

**Table 2:** Details of *Eratyrus mucronatus* collect from 1991 to 2013 in French Guiana. ^1^,^2^,^3^,^4^,^5^,^6^,^7^: different areas of French Guiana. SEAG: Société Entomologique Antilles-Guyane (Entomological Society for French West-Indies and Guiana). JMB: Jean-Michel Bérenger. BH: Bernard Hermier. CNES Kourou: Centre National d’Etudes Spatiales (National Center for Spatial Studies).

*Eratyrus mucronatus* specimens	Sex	Date of collection	Location	Area type	Sampling person	Ct values
***EmG01***	M	1998	Kaw Mountains	Primary forest—peridomestic	JMB	20.20
***EmG02***	M	1997	Kaw Montains^1^	Primary forest—peridomestic	JMB	29.64
***EmG03***	M	1998	Kaw Mountains	Primary forest—peridomestic	JMB	28.56
***EmG04***	M	1995	Nancibo^2^	Rural- peridomestic	BH	Neg
***EmG05***	M	2010	Laussat ^3^		SEAG	21.38
***EmG06***	M	2013	Horses Mountains^4^	Sylvatic	SEAG	Neg
***EmG07***	M	2013	Horses Mountains^5^	Sylvatic	SEAG	Neg
***EmG08***	M	2013	Horses Mountains^5^	Sylvatic	SEAG	19.91
***EmG09***	M	2013	Horses Mountains^5^	Sylvatic	SEAG	23.41
***EmG10***	M	2013	Horses Mountains^5^	Sylvatic	SEAG	Neg
***EmG11***	M	2013	Horses Mountains^5^	Sylvatic	SEAG	24.78
***EmG12***	F	1993	Sinnamary^6^	Urban—peridomestic	JMB	Neg
***EmG13***	M	2013	Horses Mountains^5^	Sylvatic	SEAG	13.98
***EmG14***	M	2013	Horses Mountains^5^	Sylvatic	SEAG	13.23
***EmG15***	M	1995	Degrad Kwata^7^	Sylvatic–primary forest	JMB	20.12
***EmG16***	M	1998	Kaw Mountains	Primary forest—peridomestic	JMB	19.69
***EmG17***	M	1998	Kaw Mountains	Primary forest—peridomestic	JMB	24.47
***EmG18***	M	1996	Kaw Montains^1^	Primary forest—peridomestic	JMB	Neg
***EmG19***	M	1996	Kaw Montains^1^	Primary forest—peridomestic	JMB	Neg
***EmG20***	M	2003	CNES Kourou	Savannah	JMB	Neg
***EmG21***	M	2003	CNES Kourou	Savannah	JMB	25.91
***EmG22***	M	1991	Coralie^8^	Sylvatic–secondary forest	JMB	Neg
***EmG23***	M	1993	Kaw Montains^1^	Primary forest—peridomestic	JMB	Neg

### Molecular detection

DNAs extracted from all the triatomines were included to assess the presence of *Bartonella* species. Of 23 *Eratyrus mucronatus* samples, 13 (56.5%) were positive by *Bartonella* spp.-specific qPCR, with cycle threshold (Ct) values ranging from 13.23 to 25.91 (mean: 21.44) (Table 2). These specimens were from six distinct regions and collected between 1993 and 2003. All positive specimens were male, and a majority of them were collected in sylvatic and peridomestic areas: the Kaw Mountains (38.4%) and Horses Mountains (38.4%).

All samples tested negative for the presence of *Rickettsia* spp., *Borrelia* spp. *Anaplasma* spp., *Ehrlichia* spp., *Wolbachia* spp. and *Coxiella burnetii*. *Bartonella* spp. was only detected in *Eratyrus mucronatus* specimens.

### Sequencing

**Fig 3:**
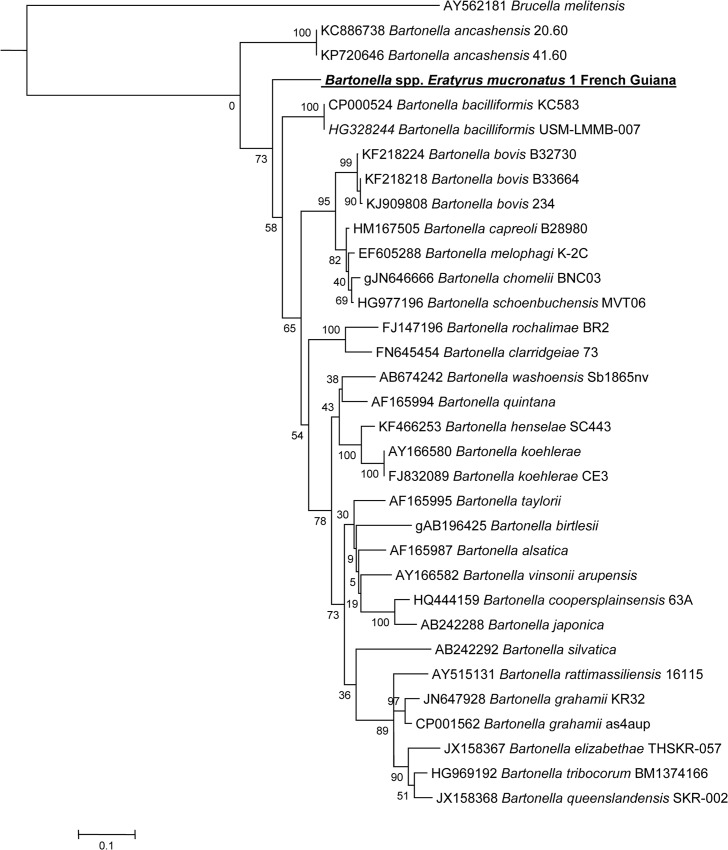
A consensus phylogenetic tree showing the relationships of the studied species of *Bartonella* species based on a portion of *rpoB* gene sequence comparison. GenBank accession numbers (or the only genome accession number) are indicated when the sequences initially originated from Genbank. The sequences were aligned using ClustalW, and phylogenetic inferences were obtained using Bayesian phylogenetic analysis with TOPALi 2.5 software (Biomathematics and Statistics Scotland, Edinburgh, UK) within the integrated Maximum Likelihood application using the TrN + I + Г model. Numbers at the nodes are percentages of bootstrap values obtained by repeating the analysis 100 times to generate a majority consensus tree. Bootstrap values below 80 were deleted from the final tree. The final set includes 756 base pairs. The new *Bartonella* sequence described in the present study is written in red.

A 787 bp fragment of the *Bartonella* spp. *rpoB* gene was amplified using conventional PCR primers prior to sequencing. Only three *ITS2*-qPCR positive samples were also positive for *rpoB* by standard PCR. Sequencing failed for two of them. Comparison of the one *rpoB* resulting sequence against the NCBI database using the BLASTN program indicated that it possessed 90% identity with the ATCC *Bartonella bacilliformis* 35685D-5 strain (#CP014012.1) and with the *B*. *bacilliformis* KC583 strain (#CP000524.1). The next closest cultivated strains were a *B*. *bovis* strain [17] and a *B*. *chomelii* strain [18], both with 89% identity. Our genotype also possesses 87% identity with a *Bartonella ancashensis* strain [19,20]. Available sequences of *Bartonella rpoB gene* were retrieved from NCBI and compared to the *Bartonella* sequence described hereby. This *Bartonella* genotype formed a distinct clade, with a strain of *Bartonella bacilliformis* as the closest clade based on *rpoB* gene analysis (Fig 3).

**Fig 4:**
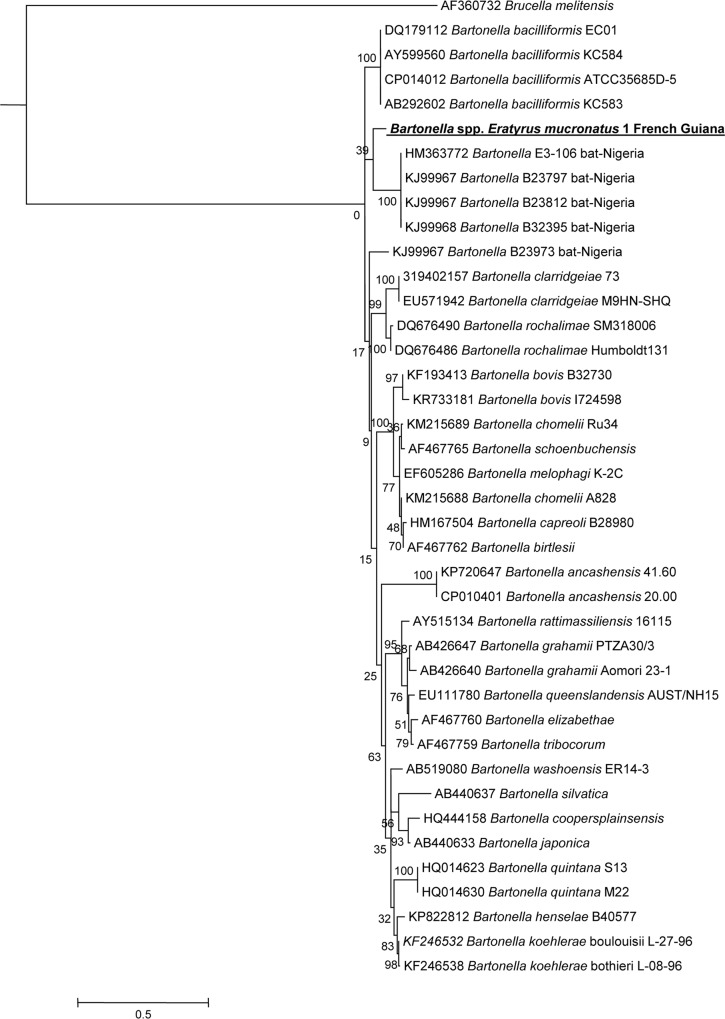
A consensus phylogenetic tree showing the relationships of the *Bartonella* species studied based on a portion of *ftsZ* gene sequence comparison. GenBank accession numbers (or the only genome accession number) are indicated when the sequences originated from Genbank at the beginning. The sequences were aligned using ClustalW, and phylogenetic inferences were obtained using Bayesian phylogenetic analysis with TOPALi 2.5 software (Biomathematics and Statistics Scotland, Edinburgh, UK) within the integrated Maximum Likelihood application using the ML SYM+I+Г model. Numbers at the nodes are percentages of bootstrap values obtained by repeating the analysis 100 times to generate a majority consensus tree. Bootstrap values below 80 were deleted from the final tree. The final set includes 292 base pairs. The new *Bartonella* sequence described in this study is written in red.

All samples allowed amplification of a single 333 bp fragment of the *ftsZ* gene by standard PCR. Blast analysis revealed 91% identity with the aforementioned 35685D-5 and KC583 *B*. *bacilliformis* strains (Fig 4).

**Fig 5:**
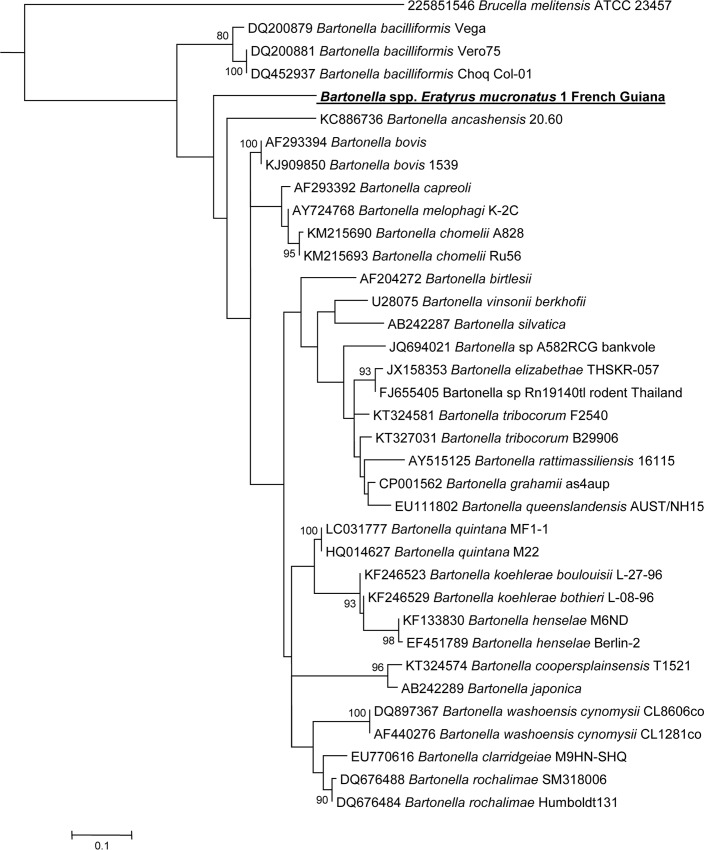
A consensus phylogenetic tree showing the relationships of the *Bartonella* species studied based on a portion of *gltA* gene sequence comparison. GenBank accession numbers (or the only genome accession number) are indicated when the sequences originated from Genbank at the beginning. The sequences were aligned using ClustalW, and phylogenetic inferences were obtained using Bayesian phylogenetic analysis with TOPALi 2.5 software (Biomathematics and Statistics Scotland, Edinburgh, UK) within the integrated Maximum Likelihood application using the K81uf + I + Г model. Numbers at the nodes are percentages of bootstrap values obtained by repeating the analysis 100 times to generate a majority consensus tree. Bootstrap values below 80 were deleted from the final tree. The final set includes 200 base pairs. The new *Bartonella* sequence described in this study is written in red.

A total of 12 out of 13 samples were successfully amplified by standard PCR targeting a fragment of the *gltA* gene. BLAST analysis showed 88% identity with uncultured *Bartonella* species detected in bank voles [21], deer [22] and bats from Africa [23], but also with *B*. *bovis and B*. *chomelii* strains (Fig 5). Based on the *gltA* gene, our genotype is 84% similar to *B*. *bacilliformis* strains. Only a single *rpoB* sequence was obtained but all *ftsZ* and *gltA* sequences obtained were identical for all *E*. *mucronatus* specimens.

**Fig 6:**
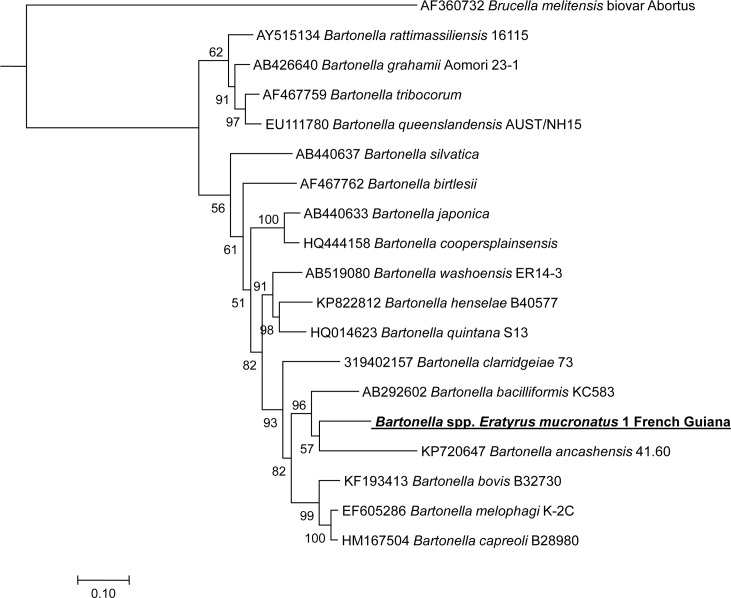
A consensus concatened phylogenetic tree showing the relationships of the *Bartonella* species studied based on a concatened sequence of *Bartonella rpoB*, *ftsZ* and *gltA* gene fragment. Concatenated *rpoB*, *ftsZ* and *gltA* sequences were aligned using CLUSTALW and phylogenetic inferences obtained using Bayesian phylogenetic analysis [Ronquist F, Huelsenbeck JP. MrBayes 3: Bayesian phylogenetic inference under mixed models. Bio-informatics 2003; 19:1572–1574] with the TOPALi 2.5 software (Biomathematics and Statistics Scotland, Edinburgh, UK) with the integrated MrBayes application [ttp://mrbayes.csit.fsu.edu] with the HKY+I+Г substitution model. GenBank accession numbers are indicated at the beginning. Numbers at the nodes are bootstrap values obtained by repeating the analysis 100 times to generate a majority consensus tree. There were a total of 1245 positions in the final dataset. The scale bar indicates a 10% nucleotide sequence divergence.

BLAST analysis of the concatened sequence of the three genes revealed 99% of coverage and 90% similarity with the two aforementioned *B*. *bacilliformis* strains. Phylogenetic analysis based on the concatened sequences revealed clustering of our *Bartonella* strain with two *B*. *bacilliformis* and *B*. *ancashensis* strains (Fig 6).

## Discussion

*Bartonella* species are small fastidious gram-negative bacteria belonging to the *Alphaproteobacteria* class that are able to infect many mammals, including humans [24]. They are mostly transmitted by arthropod vectors such as sandflies (*Lutzomyia verrucarum*), human body lice (*Pediculus humanus humanus*), different fleas including cat fleas (*Ctenocephalides felis*), biting flies and ticks [25]. Among the several *Bartonella* species, some have been identified as human pathogens, causing well-known vector-borne diseases such as Carrion’s disease (*B*. *bacilliformis)*, trench fever (*B*. *quintana)*, cat scratch disease (*B*. *henselae*) as well as endocarditis [24].

We hereby describe a novel *Bartonella* genotype, phylogenetically related to several human and animal pathogens, as shown by the phylogenetic analyses. *B*. *bacilliformis*, a closely related species, is the causative agent of the first and well-described human bartonellosis called Carrion’s disease [26]. Transmitted through the bite of an infected phlebotomine sand fly, *L*. *verrucarum*, this South American endemic bacterium can induce a biphasic illness with two distinct syndromes that can be concomitant or independent. An acute phase known as Oroya fever manifests as a hemolytic fever linked to bacteremia that can range from 10 to 210 days and can be fatal in 40–88% of individuals without treatment. The second syndrome called verruga peruana manifests as blood-filled hemangiomas due to infection of the endothelium [26]. No human cases of *B*. *bacilliformis* infection have been reported in French Guiana to date [27]. Our genotype is also closely related to a strain of *B*. *ancashensis*, a recently described *Bartonella* species closely related to *B*. *bacilliformis* that was isolated from the blood of two patients diagnosed with a chronic stage of verruga peruana in Peru [20]. All data suggest that *B*. *ancashensis* could be a second agent. Our new agent is also closely related to *B*. *bovis* strains. Isolated from cats, which are only accidental hosts, this endocarditis [28].

*E*. *mucronatus* is a sylvatic triatominae bug involved in the transmission of *T*. *cruzi* [11]. It is recognized now as an invasive species as it has been described around and inside houses since 1959 [29] because of its attraction to artificial light sources [30]. They are known to feed on bats, but also on small mammals such as xenarthrans and opossums [11]. Bats are widely reported to be sources of many viral and bacterial pathogens [31], including *Bartonella* spp. worldwide, including in French Guiana [32], Nigeria [33], Guatemala [34] and Vietnam, for example [35]. Therefore, *Bartonella* spp. were frequently detected in hematophagous arthropods feeding on bats such as bat flies (*Hippoboscidae*, *Streblidae*, *Nycteribiidae*) [36] or *Cimex adjunctus* [37]. Triatomine vectors belonging to genera *Triatoma*, *Rhodnius*, *Panstrongylus* and *Eratyrus* can be totally domiciliated or invasive, since they occasionally visit houses as described in Bolivia [38], Brazil [39], Argentina [40] and Venezuela [11]. The presence of these bugs around houses has long been known and has justified the establishment of chemical control campaigns, which after 5 years remain a failure in Bolivia [38]. The invasive behavior of *E*. *mucronatus* has not yet been described in French Guiana but data from Bolivia suggest that eliminating it once it is settled is challenging [38]. Living in various ecotopes and not host-specific [41], these bugs can easily feed both on humans and animals [38], both of them potentially bacteriemic, parasitemic or viremic at the blood meal time point.

Triatominae species are well-studied bugs, however, this work provides the first evidence to our knowledge of infection with a bacterium that is not *a priori* endosymbiotic. The specimens we analyzed were dry, with no information regarding their engorgement status at the time of collection. However, as they were collected using light traps or interception traps, we can assume that they were seeking hosts and therefore probably non-engorged. Thus, we can suppose that we did not detect DNA of a bacterium present in recently ingested blood but a genuine infection. To support this hypothesis, the infection rate was considerable (56%) among triatomines collected in very distant sampling periods, geographically and over time. Ct values were also very low, increasing the possibilities that this bacterium multiplies within the bug's body. However, to evaluate the possibility of transmission of these *Bartonella* spp., an experimental model of infection, or at least information regarding the location of the bacteria in the bug, would be necessary. Such information could not be obtained from our samples as they were dry and old. Cultivation of any bacteria or any attempt to localize with immunofluorescence, for example, was not possible.

In continuing this work, it would be interesting to collect wild *E*. *mucronatus* specimens in order to isolate the bacterium and establish an experimental model of infection with this arthropod/pathogen pair. This would reveal whether the bug is a simple carrier or an efficient vector of this *Bartonella*. The possible interaction between *T*. *cruzi* and this *Bartonella* spp. in this insect is also unknown and could be investigated by monitoring the trypanosome’s cycle and transmission in co-infected *E*. *mucronatus*. Being phylogenetically closely related to two severe human pathogens (*B*. *bacilliformis* and *B*. *ancashensis*), it would also be important to evaluate its pathogenicity. Because of the huge public health impact of Chagas disease in South America, investigations on *Triatominae* were limited to the study of their interactions with *T*. *cruzi*. In fact, Triatominae bugs may host such bacteria as *Bartonella* species and, probably, may be its vector.
